# Identification of geographically distributed sub-populations of *Leishmania *(*Leishmania*) *major *by microsatellite analysis

**DOI:** 10.1186/1471-2148-8-183

**Published:** 2008-06-24

**Authors:** Amer Al-Jawabreh, Stephanie Diezmann, Michaela Müller, Thierry Wirth, Lionel F Schnur, Margarita V Strelkova, Dmitri A Kovalenko, Shavkat A Razakov, Jan Schwenkenbecher, Katrin Kuhls, Gabriele Schönian

**Affiliations:** 1 Department of Parasitology, Institute of Microbiology and Hygiene, Charité University Medicine Berlin, Dorotheenstr. 96, D-10098 Berlin, Germany; 2 Leishmania Research Unit, Jericho, The Palestinian Authority; 3 Ecole Pratique des Hautes Etudes, Muséum National d'Histoire Naturelle, 16 rue Buffon, 72231 Paris cedex 05, France; 4 Department of Parasitology, Hebrew University-Hadassah Medical School, P. O. Box 12272, Jerusalem 91120, Israel; 5 Department of Medical Protozoology, Martsinovsky Institute of Medical Parasitology and Tropical Medicine, Sechenov Moscow Medical Academy, M. Pirogovskaya 20, 119830 Moscow, Russia; 6 Isaev Research Institute of Medical Parasitology, Department of Leishmania Epidemiology, ul Isaeva 38, 703005 Samarkand, Uzbekistan

## Abstract

**Background:**

*Leishmania *(*Leishmania*) *major*, one of the agents causing cutaneous leishmaniasis (CL) in humans, is widely distributed in the Old World where different species of wild rodent and phlebotomine sand fly serve as animal reservoir hosts and vectors, respectively. Despite this, strains of *L. (L.) major *isolated from many different sources over many years have proved to be relatively uniform. To investigate the population structure of the species highly polymorphic microsatellite markers were employed for greater discrimination among it's otherwise closely related strains, an approach applied successfully to other species of *Leishmania*.

**Results:**

Multilocus Microsatellite Typing (MLMT) based on 10 different microsatellite markers was applied to 106 strains of *L. (L.) major *from different regions where it is endemic. On applying a Bayesian model-based approach, three main populations were identified, corresponding to three separate geographical regions: Central Asia (CA); the Middle East (ME); and Africa (AF). This was congruent with phylogenetic reconstructions based on genetic distances. Re-analysis separated each of the populations into two sub-populations. The two African sub-populations did not correlate well with strains' geographical origin. Strains falling into the sub-populations CA and ME did mostly group according to their place of isolation although some anomalies were seen, probably, owing to human migration.

**Conclusion:**

The model- and distance-based analyses of the microsatellite data exposed three main populations of *L. (L.) major*, Central Asia, the Middle East and Africa, each of which separated into two sub-populations. This probably correlates with the different species of rodent host.

## Background

*Leishmania *(*Leishmania*) *major *is one of the agents causing Old World cutaneous leishmaniasis (CL) in humans, which, in the case of *L. *(*L.*) *major*, is a rural, zoonotic, vector-borne disease, involving different species of wild rodents as animal reservoir hosts and different species of phlebotomine sand flies as vectors, depending on the geographical location where it occurs (reviewed, [1]. Humans, though infected in large numbers, are considered to be incidental hosts not directly implicated in the transmission cycle [2].

The parasite and, thus, the disease are geographically widely distributed in the arid and semi-arid areas of: North-West, North, Central sub-Saharan and East Africa; the Near and Middle East; Central Asia; and Rajasthan, India. Despite the very broad geographical distribution and large variety of types of wild animal host and sand fly vector, the different strains of *L. *(*L.*) *major *isolated from many sources over many years have proved to be relatively uniform when studied by most of the classical and more modern methods used for characterizing leishmanial strains. Serological tests like excreted factor (EF) serotyping and the application of *Leishmania *species-specific monoclonal antibodies have shown that antigenic differences exist among different strains of *L. *(*L.*) *major *[3]. Multilocus enzyme electrophoresis (MLEE) exposed some enzyme electrophoretic variation with some isoenzyme variant profiles showing a degree of geographical sub-division within a general enzyme electrophoretic uniformity [4-6].

The more recent application of various molecular biological methods revealed geographically distributed genetic polymorphism among different strains of *L. *(*L.*) *major*. Analysis of sequence polymorphisms in seven coding, non-coding and anonymous nuclear DNA sequences [1] as well as a partial sequence from the kinetoplast DNA maxicircle divergent region [7] of strains of *L. *(*L.*) *major *showed that the strains from Central Asia and the Middle East were genetically distinct. None to very little variation was seen among the Central Asian strains and somewhat more among the Middle Eastern ones. Only a few East African strains of *L. *(*L.*) *major *were included in these studies, which tended to be genetically closer to the Middle Eastern than to the Central Asian ones. Different patterns in the most variable sequence were attributed to variations in complex microsatellite repeats. This prompted a search for highly polymorphic microsatellite markers that would allow greater discrimination of otherwise closely related strains of *L. *(*L.*) *major*.

Microsatellites are repeated simple motifs of a few nucleotides (<6) flanked by unique sequences. They are ubiquitous in prokaryotes and eukaryotes and have been shown to exhibit a substantial level of polymorphism in a number of eukaryotic genomes [8,9]. They are becoming one of the principal genetic marker systems in phylogenetic, population genetic and molecular epidemiological studies. The leishmanial genome is relatively rich in microsatellite sequences, about 600 (CA)_n _loci per haploid genome, with CA being the most abundant dinucleotide repeat as in all vertebrates and fungi investigated so far [9-11]. Microsatellite markers have been used successfully for characterizing and detecting genetic variation in other Old World leishmanial strains and species, i. e., *L. *(*Leishmania*) *donovani *and *L. *(*Leishmania*) *infantum *[12-14] and *L. *(*Leishmania*) *tropica *[15].

This study adds the species *L. *(*L.*) *major*. Ten informative microsatellite loci based on nucleotide sequence information of *L. *(*L.*) *major *obtained from the *Leishmania *genome project were used to determine polymorphism and micro-heterogeneity, and their geographical and epidemiological distribution.

## Results

### Microsatellite analysis

Twenty-three sequences located on chromosomes 1, 3, 5, 21 and 35 that contained nucleotide repeats such as (AT)n, (GC)n, (CA)n, (GTG)n, and (GACA)n were selected from the genome sequence of *L. *(*L.*) *major *published by the European Bioinformatics Institute. PCR primers were designed for microsatellite loci that contained at least 6 repeats in the reference strain *L. *(*L.*) *major *MHOM/IL/1980/Friedlin and were located on different chromosomes or, if located on the same chromosome, were at least distant enough to be considered unlinked (Table 1). Fifteen of the 23 primer pairs yielded a single PCR fragment for the majority of the strains tested. Ten of these 15 primer pairs amplified polymorphic fragments of different size in different strains of *L. *(*L.*) *major *(Table 1).

**Table 1:** The microsatellite markers used.

Marker	Size (bp)	Primer sequences	T_A_	Chromosome	Location at bp	Repeat number*	No. of alleles
4GTG	70	F: 5'cggtttggcgctgaaagcgg R: 5'cgtgaggacgccaccgaggc	58°C	35	6460-530	7	3
27GTG	75	F: 5'ggaggtggctgtggttgttg R: 5'gccgctgacgctgcaggct	58°C	3	1440-515	9	3
36GTG	68	F: 5'agcgaagaagagtcgggcag R: 5'gcgccttcagtgcgtcgtcc	62°C	1	140895-963	9	4
39GTG	86	F: 5'gtcttgccgcgaggtgaccg R: 5'ccagcaccagcaccaccatc	58°C	1	202765-851	9	5
45GTG	86	F: 5'acggccgggtggtcgtgggt R: 5'cgttcgcacgcacgcacgca	58°C	1	59751-890	12	7
1GC	64	F: 5'ctggcacgcacacccacaca R: 5'atctgcgctcatctggcgag	60°C	3	10323-386	7	2
28AT	65	F: 5'ttgcctatcaacacaaggct R: 5'agtctctctctctctctata	42°C	5	27966-031	9	6
71AT	55	F: 5'tcttgcgaaggtgtggtctt R: 5'agcccacgtgtacatgtgtg	50°C	21	22113-168	13	9
1GACA	75	F: 5'gaaagggcaggaggacggat R: 5'cacacacacatacacacata	54°C	1	68459-534	7	3
1CA	87	F: 5'ttagttccatcatacacccg R: 5'cgttcgacatggagaataag	48°C	35	30151-238	14	7

* in *L. *(*L.*) *major *MHOM/IL/1980/Friedlin

T_A _annealing temperaturea

Only 4 out of 45 pair-wise combinations (9%) were in significant linkage disequilibrium (*P *< 0.05) when GENEPOP was used. No linkage was observed using the Bonferroni corrections implemented in FSTAT.

The 10 polymorphic loci were used to perform multi-locus microsatellite typing (MLMT) on 106 strains of *L. *(*L.*) *major *collected in 10 Asian and 9 African countries. Four of the 10 loci showed only homozygous allele combinations. However, three GTG loci, two AT loci and the GACA locus were heterozygous for one or more of the strains (see Additional file 1). In some cases, no PCR product could be obtained in repeated PCR runs, which was treated as missing data for the statistical analyses.

**Figure 1:**
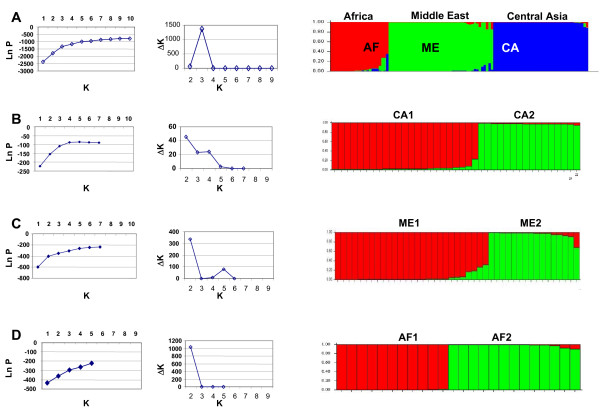
**Estimated population structure obtained with STRUCTURE**. This is shown as plots of the estimated membership coefficient (Q), which is represented by a single vertical line for each sample. Coloured segments represent the sample's estimated membership in each of the K inferred clusters. Individual isolates can have membership in multiple clusters, with membership coefficients summing up to 1 across clusters. Panel A represents the STRUCTURE result for the whole data set, whereas panels B – D show sub-structuring obtained when STRUCTURE was re-run for each main population separately. CA = Central Asia; ME = Middle East; AF = Africa.

**Figure 2:**
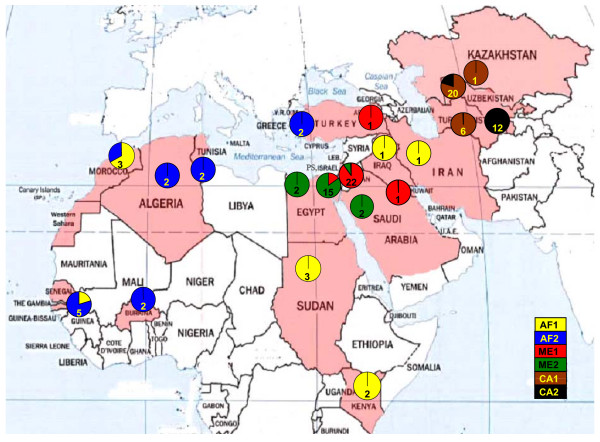
**Distribution of different sub-populations of *L. *(*Leishmania*) *major *in the three main geographical regions**. Populations CA1, CA2, ME1, ME2, AF1 and AF2 are labelled with different colours. The percentage of strains from a given focus belonging to these populations is shown as a pie diagram, which also shows the number of strains collected there. PS = The Palestinian Authority.

**Figure 3:**
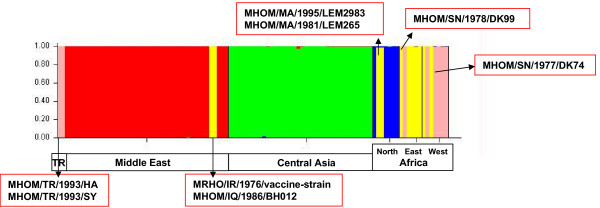
**Population structure as shown by plots of Q (estimated membership coefficient for each sample), using five populations pre-defined according to their geographical origin**. Regions may have more than one colour. For instance, strains from Iran and Iraq geographically belong to ME but genetically to East Africa as represented by the colour yellow.

Sixty-six differing MLMT profiles comprising the number of repeats in each of the 10 markers were revealed among the 106 strains (see Additional file 1). The MLMT profiles were named Lmj, thus indicating that these microsatellite profiles are unique to *L. *(*L.*) *major*, and numbered from 1 to 66. Figures 1 to 3 show their genetic inter-relationship. Most profiles were represented by a single strain; eleven were present in more than one strain. Nine microsatellite variant profiles were exposed among the 23 strains in the sub-population CA1; six among the 16 strains in the sub-population CA2; thirteen among the 26 strains in the sub-population ME1; fifteen among the 17 strains in the sub-population ME2; eleven among the 11 strains in the sub-population AF1; and twelve among the 13 strains in the sub-population AF2 (see Table 2, and Figure 2 for origins and geographical distributions).

**Table 2:** The strains of *Leishmania *(*L.*) *major *analysed in this study.

WHO code	Origin country, location, source	Microsatellite profile	Population	General region
MHOM/TM/1973/5ASKH	Turkmenistan, Ashkhabad: Human	Lmj 01	CA1	Central Asia (CA)
MHOM/TM/1987/Rod	Turkmenistan, Bakharden: Human	Lmj 01	CA1	
IPAP/TM/1991/M-97	Turkmenistan, Tezeel: *Ph. papatasi*	Lmj 01	CA1	
MRHO/TM/1995/T-9537	Turkmenistan, Serags:*R. opimus*	Lmj 02	CA1	
MHOM/TM/1982/Lev	Turkmenistan, Geok-Depe: Human	Lmj 03	CA1	
MHOM/TM/1986/ER	Turkmenistan, Tejen: Human	Lmj 04	CA1	
MRHO/KZ/1988/Tur-27R	Kazakhstan, Turkestan:*R. opimus*	Lmj 01	CA1	
MHOM/UZ/1987/BUR	Uzbekistan, Karaulbasar: Human	Lmj 01	CA1	
MRHO/UZ/1987/KK29	Uzbekistan, Takhtakupyr: *R. opimus*	Lmj 01	CA1	
MHOM/UZ/1987/Kurb	Uzbekistan, Karshi: Human	Lmj 02	CA1	
MHOM/UZ/1999/Nuriya	Uzbekistan, Mubarak: Human	Lmj 01	CA1	
MHOM/UZ/2002/Isv M-22h	Uzbekistan, Mubarak: Human	Lmj 05	CA1	
MHOM/UZ/2002/Isv M-17h	Uzbekistan, Mubarak: Human	Lmj 06	CA1	
MHOM/UZ/2002/Isv M-12h	Uzbekistan, Mubarak: Human	Lmj 06	CA1	
MHOM/UZ/2002/Isv M-30h	Uzbekistan, Mubarak: Human	Lmj 06	CA1	
MHOM/UZ/2002/Isv M-28h	Uzbekistan, Mubarak: Human	Lmj 06	CA1	
MHOM/UZ/2002/Isv M-25h	Uzbekistan, Mubarak: Human	Lmj 06	CA1	
MHOM/UZ/1998/Isv M-09h	Uzbekistan, Mubarak: Human	Lmj 06	CA1	
MHOM/UZ/1998/Isv M-01h	Uzbekistan, Mubarak: Human	Lmj 06	CA1	
MHOM/UZ/2002/Isv M-10h	Uzbekistan, Mubarak: Human	Lmj 07	CA1	
MHOM/UZ/2002/Isv M-29h	Uzbekistan, Mubarak: Human	Lmj 08	CA1	
MHOM/UZ/1998/Isv M-04h	Uzbekistan, Mubarak: Human	Lmj 08	CA1	
MHOM/UZ/1998/Isv M-08h	Uzbekistan, Mubarak: Human	Lmj 09	CA1	
				
MHOM/UZ/1998/Isv M-02h	Uzbekistan, Mubarak: Human	Lmj 13	CA2	
MHOM/UZ/2002/Isv M-27h	Uzbekistan, Mubarak: Human	Lmj 10	CA2	
MHOM/UZ/2002/Isv M-26h	Uzbekistan, Mubarak: Human	Lmj 11	CA2	
MRHO/UZ/1959/NealP	Uzbekistan, Karakul: *R. opimus*	Lmj 14	CA2	
MHOM/UZ/2000/Isv T-03h	Uzbekistan, Termez: Human	Lmj 12	CA2	
MHOM/UZ/2003/Isv T-21h	Uzbekistan, Termez: Human	Lmj 15	CA2	
MHOM/UZ/2003/Isv T-24h	Uzbekistan, Termez: Human	Lmj 15	CA2	
MHOM/UZ/2003/Isv T-29h	Uzbekistan, Termez: Human	Lmj 15	CA2	
MHOM/UZ/2003/Isv T-32h	Uzbekistan, Termez: Human	Lmj 15	CA2	
MHOM/UZ/2003/Isv T-35h	Uzbekistan, Termez: Human	Lmj 15	CA2	
MRHO/UZ/2003/Isv T-6g	Uzbekistan, Termez: *R. opimus*	Lmj 15	CA2	
MRHO/UZ/2003/Isv T-20g	Uzbekistan, Termez: *R. opimus*	Lmj 15	CA2	
MRHO/UZ/2003/Isv T-23g	Uzbekistan, Termez: *R. opimus*	Lmj 15	CA2	
MRHO/UZ/2003/Isv T-38g	Uzbekistan, Termez:*R. opimus*	Lmj 15	CA2	
MRHO/UZ/2003/Isv T-44g	Uzbekistan, Termez: *R. opimus*	Lmj 15	CA2	
MRHO/UZ/2003/Isv T-37g	Uzbekistan, Termez: *R. opimus*	Lmj 15	CA2	
				
MHOM/MA/1995/LEM2983	Morocco, Er Rachidia: Human	Lmj 18	AF1	Africa (AF)
MHOM/MA/1981/LEM265	Morocco, Tata: Human	Lmj 19	AF1	
MHOM/SN/1977/DK74	Senegal, M'bour: Human	Lmj 20	AF1	
MHOM/SD/2004/MW1	Sudan, location not known: Human	Lmj 22	AF1	
MHOM/SD/2004/MW38	Sudan, location not known: Human	Lmj 23	AF1	
MHOM/SD/2004/MW94	Sudan, location not known: Human	Lmj 24	AF1	
MTAT/KE/195?/T4	Kenya, Baringo:*Tatera *sp.	Lmj 21	AF1	
MTAT/KE/????/NLB089A	Kenya, Marigat:*T. robusta*	Lmj 17	AF1	
MHOM/??/1987/NEL2	Africa, location not known	Lmj 16	AF1	
MHOM/IQ/1986/BH012	Iraq, location not known: Human	Lmj 38	AF1	
MRHO/IR/1976/vaccine-strain	Iran, location not known: *R. opimus*	Lmj 37	AF1	
				
MHOM/MA/1992/LEM2463	Morocco, Ain Beni Mathar: Human	Lmj 25	AF2	
MHOM/DZ/1998/CRE95	Algeria, M'sila: Human	Lmj 26	AF2	
MHOM/DZ/1998/LPS13	Algeria, Biskra: Human	Lmj 27	AF2	
MHOM/TN/1997/LPN162	Tunisia, Sfax: Human	Lmj 34	AF2	
MHOM/TN/1994/GLC7	Tunisia, Gafsa: Human	Lmj 28	AF2	
MHOM/SN/1978/DK106	Senegal, Djourbel: Human	Lmj 30	AF2	
MHOM/SN/1996/DPPE23	Senegal, Thies: Human	Lmj 32	AF2	
MHOM/SN/1996/LEM3181	Senegal, Thies: Human	Lmj 31	AF2	
MHOM/SN/1978/DK99	Senegal, Matam: Human	Lmj 29	AF2	
MHOM/BF/1996/LIPA538	Burkino Faso, Ouagadougou: Human	Lmj 33	AF2	
MHOM/BF/1998/LPN166	Burkino Faso, Ouagadougou: Human	Lmj 35	AF2	
MHOM/TR/1993/HA	WesternTurkey, Manisa: Human	Lmj 36	AF2	
MHOM/TR/1993/SY	WesternTurkey, Aydin: Human	Lmj 36	AF2	
				
MHOM/TR/1994/HK	Eastern Turkey, Kars: Human	Lmj 50	ME1	Middle East (ME)
MHOM/PS/1998/ISLAH388	The Palestinian Authority, Jericho: Human	Lmj 39	ME1	
MHOM/PS/1998/ISLAH389	The Palestinian Authority, Jericho: Human	Lmj 40	ME1	
MHOM/PS/1998/ISLAH402	The Palestinian Authority, Jericho: Human	Lmj 40	ME1	
MHOM/PS/2000/ISLAH503	The Palestinian Authority, Jericho: Human	Lmj 39	ME1	
MHOM/PS/2000/ISLAH506	The Palestinian Authority, Jericho: Human	Lmj 40	ME1	
MHOM/PS/2001/ISLAH600	The Palestinian Authority, Jericho: Human	Lmj 49	ME1	
MHOM/PS/2001/ISLAH659	The Palestinian Authority, Jericho: Human	Lmj 40	ME1	
MHOM/PS/2001/ISLAH657	The Palestinian Authority, Jericho: Human	Lmj 41	ME1	
MHOM/PS/2001/ISLAH658	The Palestinian Authority, Jericho: Human	Lmj 42	ME1	
MHOM/PS/2002/ISLAH662	The Palestinian Authority, Jericho: Human	Lmj 43	ME1	
MHOM/PS/2002/ISLAH697	The Palestinian Authority, Jericho: Human	Lmj 44	ME1	
MHOM/PS/2002/ISLAH690	The Palestinian Authority, Jericho: Human	Lmj 44	ME1	
MHOM/PS/2002/ISLAH691	The Palestinian Authority, Jericho: Human	Lmj 45	ME1	
MHOM/PS/2003/ISLAH718	The Palestinian Authority, Jericho: Human	Lmj 47	ME1	
MHOM/IL/1967/Jericho II	Israel, Jericho: Human	Lmj 39	ME1	
MHOM/IL/1986/Blum	Israel, Jericho: Human	Lmj 39	ME1	
MHOM/IL/1990/LRC-L585	Israel, Jericho: Human	Lmj 39	ME1	
MHOM/IL/1980/Friedlin	Israel, Almog: Human	Lmj 39	ME1	
MHOM/IL/2003/LRC-L949	Israel, Nabi Musa: Human	Lmj 46	ME1	
MHOM/IL/2003/LRC-L964	Israel, near Beersheba: Human	Lmj 48	ME1	
MHOM/IL/2003/LRC-L965	Israel, Qeziot: Human	Lmj 39	ME1	
MHOM/IL/2003/LRC-L962	Israel, location not known: Human	Lmj 48	ME1	
MPSA/IL/1983/PSAM398	Israel, Arava Hatzeva: *Ps. obesus*	Lmj 39	ME1	
MHOM/IL/2000/LRC-L779	Israel, Arava, Ein Yahav, or Egypt, Sinai:Human?	Lmj 39	ME1	
MHOM/KW/1976/P47	Kuwait, location not known: Human	Lmj 51	ME1	
				
MHOM/PS/2003/ISLAH720	The Palestinian Authority, Jericho: Human	Lmj 52	ME2	
MHOM/IL/2003/LRC-L1000	Israel, Qalya, northern Dead Sea: Human	Lmj 64	ME2	
MHOM/IL/2003/LRC-L960	Israel, Revivim: Human	Lmj 62	ME2	
MHOM/IL/2001/LRC-L846	Israel, Yerucham: Human	Lmj 55	ME2	
MHOM/IL/2002/LRC-L946	Israel, Yerucham: Human	Lmj 58	ME2	
MHOM/IL/2002/LRC-L940	Israel, Yerucham: Human	Lmj 59	ME2	
MHOM/IL/2003/LRC-L963	Israel, Shifta: Human	Lmj 63	ME2	
MHOM/IL/2003/LRC-L952	Israel, Qeziot: Human	Lmj 57	ME2	
MHOM/IL/2002/LRC-L862	Israel, near Mitzpe Ramon: Human	Lmj 61	ME2	
MHOM/IL/2003/LRC-L958	Israel, En Yahav: Human	Lmj 60	ME2	
IPAP/IL/1984/LRC-L465	Israel, Uvda: *Ph. papatasi*	Lmj 53	ME2	
IPAP/IL/1998/LRC-L746	Israel, Uvda: *Ph. papatasi*	Lmj 53	ME2	
IPAP/IL/1984/LRC-L464	Israel, Uvda: *Ph. papatasi*	Lmj 54	ME2	
MHOM/EG/1984/LRC-L505	Egypt, Sinai: Human	Lmj 56	ME2	
IPAP/EG/1989/RTC-13	Egypt, Sinai: *Ph. papatasi*	Lmj 53	ME2	
MHOM/SA/1984/KFUH68757	Saudi Arabia, Hofuf?: Human	Lmj 66	ME2	
MPSA/SA/1989/SABIR-1	Saudi Arabia, Hofuf: *Ps. obesus*	Lmj 65	ME2	

Key: *Ph*. = *Phlebotomus*; *R*. = *Rhombomys*; *T*. = *Tatera*; *Ps*. = *Psammomys*; The prefix Lmj indicates that these microsatellite profiles are unique to *L*. (*L.*) *major*.

Table 3 shows that the number of alleles varies between loci, ranging from 3 to 10. An increased degree of inbreeding within loci (*F*_*is*_) was detected, ranging from 0.874–1.00 (*P *< 0.05) with a mean of 0.976. Observed heterozygosity (*H*_*o*_) among loci was extremely low compared with the heterozygosity (*H*_*e*_) expected under assumption of the Hardy-Weinberg equilibrium. These results clearly show that *L. *(*L.*) *major *is largely clonal and that hybridization only occurs at very low frequencies.

**Table 3:** Population genetic statistics of the microsatellite markers.

Locus	Descriptive statistics				
	*N*	*A*	*He*	*Ho*	*Fis*

4GTG	105	3	0.362	0.0095	0.974
27GTG	105	6	0.631	0.000	1.000
36GTG	101	7	0.711	0.000	1.000
39GTG	93	7	0.737	0.011	0.985
45GTG	104	10	0.784	0.000	1.000
1GC	103	3	0.330	0.000	1.000
28AT	104	8	0.533	0.029	0.946
71AT	105	10	0.604	0.076	0.874
1GACA	103	3	0.548	0.009	0.982
1CA	102	10	0.509	0.000	1.000

All	102.5	6.7	0.575	0.014	0.976

The average sample size (*N*), average number of alleles per locus (*A*), expected proportion of heterozygotes (*He*), observed proportion of heterozygotes (*Ho*) (by Hardy-Weinberg equilibrium) and inbreeding coefficient (*Fis*) were obtained using GDA software. LD was calculated using GENEPOP and FSTAT software.

### Population structure

The population structure was investigated, using the Bayesian-model based clustering approach implemented in the software STRUCTURE [16]. The most probable number of populations in this data set by calculating ΔK was three: Central Asia (CA, comprising strains from Uzbekistan, Turkmenistan and Kazakhstan; the Middle East (ME), comprising strains from Israel, The Palestinian Authority, Egypt, Saudi Arabia, Kuwait and eastern Turkey; and Africa (AF), comprising strains from North, East and West Africa, western Turkey, Iran and Iraq (Figure 1A and Table 2).

STRUCTURE analyses were re-done with each of the three main populations, CA, ME and AF, to expose further sub-division; and each main population did separate into two sub-populations: CA1 and CA2; ME1 and ME2; AF1 and AF2 (Figures 1B, C and 1D, Table 2). Although calculations of ΔK do not allow for differentiation between one or two groups in the dataset, the strains' assignment (Table 2) was most congruent with the existence of two sub-populations. The distribution of the different sub-populations is shown in Figure 2.

On using a predefined clustering approach, the total area from which the strains of *L. *(*L.*) *major *were collected was divided into five geographical sectors: Central Asia (CA), the Middle East (ME), North, West and East Africa with the last three being referred to collectively as Africa (AF). The boundaries between these sectors are mainly significant geographical barriers like seas and deserts. Each strain was assigned to one of the sectors, according to its place of collection without regard to underlying genetic relationships (Figure 3). The Central Asian cluster was highly homogenous, genetically and according to predefined geographical placement, with all the strains having a very high membership coefficient of 0.999. The Middle East cluster was also genetically well defined, except for the exclusion of the two western Turkish and sole Iraqi and Iranian strains, which grouped with the African strains. The entire African cluster was very heterogeneous regarding MLMT profiles and did not separate into distinct North, West and East African regional clusters as envisaged by the predefined clustering approach.

Increasing values of K (2–6) were used to identify ancestral populations. At K = 2, the first split separated the population CA from other strains. At K = 3, three main populations, CA, ME and AF were exposed. At K = 4, the African strains split into two clusters. At K = 5, the Middle Eastern cluster separated into two distinct sub-clusters, ME1 and ME2 while at K = 6 the population CA split into the sub-clusters CA1 and CA2.

### Construction of distance trees

**Figure 4:**
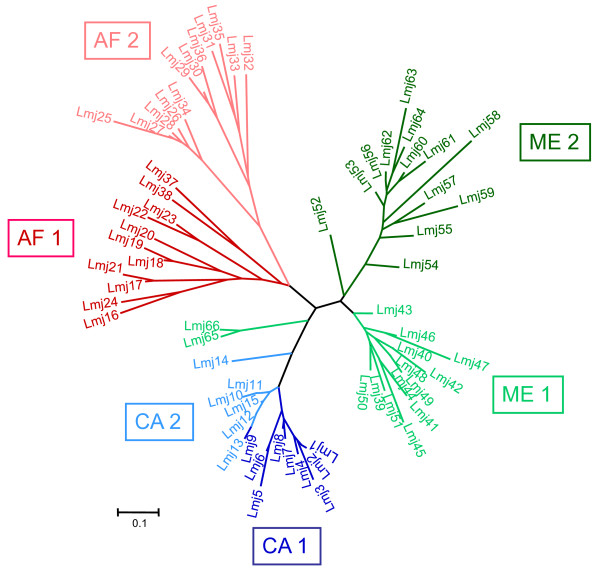
**Unrooted neighbour-joining tree calculated from genetic distances (Dps) for the 66 microsatellite profiles of *L. *(*Leishmania*) *major***. Strains representing the different microsatellite profiles are listed in Table 2. Populations and sub-populations as inferred by STRUCTURE are indicated.

For the phylogenetic analyses of these strains of *L. *(*L.*) *major*, the dataset was clone-corrected and presented 66 microsatellite genotypes. NJ and UPMGA trees were constructed from the different distance matrices obtained. All phylograms displayed the same five clusters, albeit with different bootstrap support values, as shown, for example, in the unrooted NJ tree (Figure 4) that was based on a Dps distance matrix and has: i) a Central Asian cluster; ii) two clusters of strains from the Middle East, corresponding to the sub-populations ME1 and ME2 as determined by STRUCTURE, and iii) two clusters of strains from Africa, which correlate perfectly with the sub-populations AF1 and AF2 as determined by STRUCTURE. The sub-division seen in the Central Asian cluster is largely congruent with the two sub-populations CA1 and CA2 identified by STRUCTURE. As in the STRUCTURE analysis, the two western Turkish strains (Lmj 36) grouped with the strains in the sub-population AF2 while the eastern Turkish one (Lmj 50) belonged to the sub-population ME 1. The two strains from Saudi Arabia formed a unique branch in the unrooted NJ tree, closer to the Central Asian cluster, however with only low bootstrap values.

### Population genetic data

*Fst*, as a measure of genetic differentiation between populations, calculated in a pair-wise manner for the main populations, revealed high genetic differentiation between the populations CA and ME, and CA and AF. Genetic isolation was less pronounced between the populations ME and AF. When *Fst *was calculated at a sub-population level, moderate (*Fst *= 0.05 – 0.15), high (*Fst *= 0.15 – 0.25) and very high (*Fst *> 0.25) genetic differentiation was observed for the pairs AF1/AF2, ME1/ME2 and CA1/CA2, respectively (Table 4).

**Table 4:** Estimates of F-statistics (*Fst*), measures of genetic differentiation, and migration rates (Nm) for all loci studied among the populations of *Leishmania *(*Leishmania*) *major *measured by FSTAT.

Populations	Fst	Migration rate (Nm)
CA vs. ME	0.3715	0.4220
CA vs. AF	0.5156	0.2348
ME vs. AF	0.1730	1.1950

CA1 vs. CA2	0.2936	0.6014
ME1 vs. ME2	0.1565	1.3474
AF1 vs.AF2	0.1043	2.1469

Genetic flow or migration rate, Nm, refers to the movement of strains between populations and sub-populations and sets a limit as to how much genetic divergence can occur. At the sub-population level, the genetic flow was the highest between the two African sub-populations and the least between the two Central Asian sub-populations (Table 4). At the pre-defined population classification, there was clear genetic flow (Nm) between the African regions (1.4962 and 1.7216).

### The numbers of alleles encompassed by geographical groupings

To compare the number of alleles falling within the three main geographical groupings, the MLMT profiles were grouped according to their geographical origin as specified by their WHO codes: Central Asia (15 profiles); the Middle East (27 profiles); and Africa (21 profiles). In order to compare groups of equal size, a re-sampling procedure was used [15]. The African group had the highest number of alleles (20) followed by Middle Eastern group (17) and then the Central Asian group (12).

## Discussion

Elfari et al. [1] showed that genetic and biological variation among strains of *L. *(*L.*) *major *tended to correspond with their geographical origin. Among the several analytical techniques and methods developed in that study, only two employed genetic markers that were polymorphic enough to distinguish between closely related strains. For population genetic studies of *L. *(*L.*) *major *like this one, highly discriminative markers had to be developed. Of the microsatellite markers designed, ten, all based on sequences published by the *Leishmania *Genome Project [11], have proved to be suitable for multilocus microsatellite typing (MLMT). Microsatellite analysis of the 106 strains of *L. *(*L.*) *major *revealed 66 different microsatellite profiles. These were unique to this species of *Leishmania *and demonstrated substantial genetic micro-heterogeneity with regard to microsatellite profiles. In many cases in this study, particular microsatellite profiles were found in single strains. Had many more strains of *L. *(*L.*) *major *been available, these profiles would, probably, have been associated with groups of strains as was seen with the strains isolated at Mubarak and Termez, Uzbekistan, (Table 2 and Figure 3).

The model- and distance-based analyses of the microsatellite data exposed three main populations of *L. *(*L.*) *major*, corresponding to three separate geographical regions, Central Asia, the Middle East and Africa. When the data were analyzed by STRUCTURE with an increase in the number of populations from 2 to 6 in order to identify the ancestral source population, the first split separated the Central Asian strains of *L. *(*L.*) *major *from all the others, suggesting, possibly, their older origin. The Bayesian algorithm implemented in STRUCTURE was more appropriate for characterizing population structure because it identified distinct sub-populations based on patterns of allele frequencies and, also, determined fractions of the genotype that belong specifically to each sub-population. STRUCTURE has been shown to accurately infer individual ancestries [16] and provide information on population relationships and history [17].

The presence of these three populations was supported by the detection of genetic isolation among them, particularly between population CA and the two populations AF and ME that were separate but genetically closer to one another. This may reflect the geographical distance and barriers between the three main clusters (data not shown).

**Figure 5:**
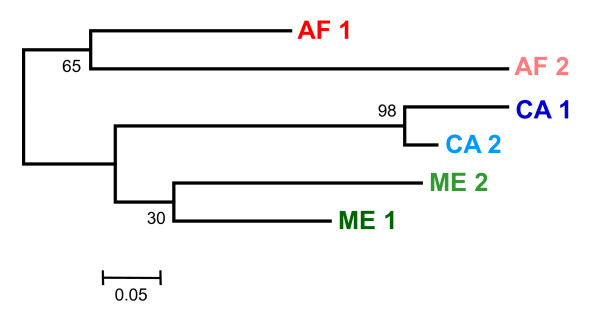
**Genetic distances between populations pre-defined by *Structure***. Strains belonging to populations and sub-populations as inferred by *Structure *are indicated in Table 2.

Both types of analysis led to sub-division of the main clusters. Congruence of the results supported tripartite clustering despite the absence of strong bootstrap values on the main branches of the distance tree (Figures 4 and 5). The two sub-groups in each of the three main populations were also supported by F-statistics, although the genetic differentiation was less pronounced, especially between the sub-populations AF1 and AF2, and between the sub-populations ME1 and ME2. Wright (1978) maintained that even slight genetic differentiation among sub-populations is significant.

Within the context of this study, it seems that the populations AF and ME were more genetically diversified than the population CA. This might be due to different sampling procedures in the three areas. The 22 strains from Africa were collected between 1976 and 2004 in 16 separated locations. In contrast to that, the 37 ME strains were collected between 1967 and 2003 from different foci encompassed by the Eastern Mediterranean region and Arabian peninsula, and the 39 strains from Central Asia between 1973 and 2003 in only 3 countries. To correct, at least partially, for this sampling bias mean number of alleles have been calculated based on the MLMT profiles present in the three areas, e.g. by excluding profiles shared by more than one strain. When corrected for Central Asia, the smallest group, the lowest number of alleles was still found in Central Asia followed by the Middle East and then Africa. The sampling bias might still account for the high heterogeneity of the African strains, but the differences in diversity for CA and ME strains cannot be explained only by differences in sampling. Most of the ME strains were collected in Israeli and Palestinian foci from a territory much smaller than the Central Asian area. The area from where the Central Asian strains of *L. *(*L.*) *major *studied here came is a landlocked generally arid plateau. The greater variation of habitats and biotopes within Middle Eastern environments seems to translate into a greater variety of animal hosts and sand fly vectors compared to Central Asia.

Where groups of strains with the same microsatellite profile were available, it was possible to make some assessment with regard to time and spatial distribution. For example, the strains in the groups of strains with the microsatellite profiles Lmj 6, Lmj 15 and Lmj 40 were isolated in a given area for each group at the same time or within short periods of time. However, strains with the microsatellite profile Lmj 1, Lmj 39 and Lmj 53 were isolated in several locations at different times. This, in addition to the observed low heterozygosity at microsatellite loci, seems to provide evidence against extensive and frequent genetic exchange within *L. *(*L.*) *major*. That microsatellite variants can persist for long periods of time and are restricted or widely spread in their distribution, supposedly, results from the effect of biotopic conditions on the animal hosts and vectors of leishmanial parasites and much less so in the case of the humans, who do alter the biotope by construction, agriculture and many of their other activities.

Most of the strains in the population ME came from Israel and The Palestinian Authority and their separation into the sub-populations ME1, Jordan Valley-Dead Sea area, and ME2, the Negev-Sinai area, did seem to parallel geographical distribution but not entirely so. Most 'misplaced' strains were from human cases and could be explained by travel and migration between these areas. Several strains have membership coefficients for both sub-populations, indicating gene flow between them.

The two African sub-populations did not correlate well with the geographical origin of the strains that fell into them. Their analysis was hampered owing to the small number of strains available and their very wide geographical dispersal. Unlike Dps-based phenetic analysis (NJ, UPGMA), which is not affected by population size [18], STRUCTURE improves with increasing sample size for each population [16]. In this study, the results obtained using STRUCTURE were consistent with those of the phenetic analysis. Nevertheless, more strains from different African foci need to be analysed to really determine the population structure of African strains of *L. *(*L.*) *major*. Preferably, one should use strains isolated from sand fly vectors and rodent hosts to avoid the effect of human migration. Small sample size was also a problem in the case of the two single strains from Iran and Iraq and the two from western Turkey that grouped together with the African strains and the eastern Turkish strain that grouped with the Middle Eastern strains (Table 2 and Figure 3).

Waki et al. [19] hypothesized that adaptation to the micro-environment in the macrophages of the mammalian hosts, the place of long-term residence of all leishmanial parasites, including *L. *(*L.*) *major*, is most significant in the evolution of the genus *Leishmania*. They considered adaptation to the sand fly vectors' gut, the place of short-term residence, to be of lesser evolutionary importance. That the three main populations of *L. *(*L.*) *major *identified by this study exist in endemic foci that each have different rodent species serving as the reservoir hosts [20] seems to support this hypothesis. Variation among the strains of *L. *(*L.*) *major *from the same endemic area leading to different sub-populations could be attributed to differences in sand fly vector populations. For instance, analysis of mitochondrial cytB haplotypes exposed two genetically distinct populations of *Ph. papatasi *in the Middle East [21].

## Conclusion

Multilocus Microsatellite Typing (MLMT) based on 10 different microsatellite markers and using a Bayesian model-based analysis as well as phylogenetic reconstructions based on genetic distances, identified three main populations, corresponding to three separate geographical regions: Central Asia (CA); the Middle East (ME); and Africa (AF). Each of them separated into two sub-populations which might reflect the existence of different species of rodent host in these areas. The African and Middle Eastern populations seemed to be more genetically diversified than the Central Asian population. Sampling bias might account for the high heterogeneity of the African strains but cannot explain the differences in diversity for Central Asian and Middle Eastern strains.

## Methods

### The parasites

Table 2 lists the 106 strains of *L. *(*L.*) *major *used. They represented almost the whole geographical range of the species. No strains from Afghanistan and Rajasthan, India, were available.

Most of them were from the collections kept in The Department of Parasitology of Hebrew University Jerusalem, The *Leishmania *Research Unit, The Islah Clinic, Jericho, The Isaev Research Institute of Medical Parasitology Samarkand, The Martsinovsky Institute of Medical Parasitology, Moscow, and The *Leishmania *Reference Centre, CHU of Montpellier.

Cultivation of the parasites and extraction of DNA were done according to [22]. DNA of MHOM/WA/87/NEL2, a strain isolated from a patient who had travelled in North, West and East Africa, was obtained from Dr. H. Schallig of the Royal Tropical Institute Amsterdam, and DNA from three Sudanese strains from Dr. M. Mukhtar of the Institute of Endemic Diseases, University of Khartoum. DNA from the three Turkish strains [23] was provided by Dr. K.P. Chang of the Department of Microbiology/Immunology, Chicago Medical School, Chicago, IL, USA.

### Microsatellite loci

Microsatellite loci were identified by searching the nucleotide sequence information assembled by the '*Leishmania major *Friedlin Genome project [24]. PCR primers potentially suited for locus-specific amplification were designed by deducing 20-nucleotide long primers from sequences precisely 5 nucleotides upstream and downstream of the repeat. The primer sequences and their annealing temperatures are listed in Table 1.

Amplification was performed in volumes of 50 μl. Twenty ng DNA were added to a PCR mixture containing: 200 μM of each dNTP; 1.5 mM MgCl_2_; 1 U *Taq *polymerase (Perkin-Elmer), and 20 pmol of forward primer and 15 pmol of reverse primer. Samples were overlaid with sterile, light mineral oil and amplified in a Robocycler Gradient 40 (Stratagene) with initial denaturation at 95°C for 6 min followed by 35 cycles of denaturation at 94°C for 30 sec, annealing at the specific primer annealing temperature (Table 1) for 30 sec and extension at 72°C for 1 min. This was followed by a final extension cycle at 72°C for 10 min. DNA of *L. *(*L.*) *major *MHOM/IL/1980/Friedlin was amplified in each experiment as a positive control.

### Microsatellite electrophoretic analysis

Amplification products were analyzed using polyacrylamide gel electrophoresis (PAGE), or MetaPhor agarose (Cambrex Bio Science Rockland, Inc, Rockland ME, USA) gel, or capillary electrophoresis. The strain *L. *(*L.*) *major *MHOM/IL/1980/Friedlin was included in every experiment. For PAGE, 15–25 μl of the PCR product were mixed with loading buffer and separated in 12% polyacrylamide gel under non-denaturating conditions at a constant voltage of 1000 V for 4 h or at 8–10 W overnight. Gels were silver-stained and dried as described by Lewin et al. [25]. Screening for microsatellite variation was also done using 3.5% MetaPhor agarose gels [13]. The lengths of PCR products in PAGE and Metaphor agarose electrophoresis were determined by comparing them with small-molecular-size markers (10-bp ladder; Invitrogen, Life Tech, Carlsbad, CA USA). Fluorescent-labelled PCR products were analyzed and measured for size with the fragment analysis tool of the CEQ 8000 automated genetic analysis system (Beckman Coulter, USA) as previously described [13,26]. The fragment sizes estimated by the different methods used were always compared to that of strain *L. *(*L.*) *major *MHOM/IL/1980/Friedlin. Repeat numbers were calculated based on the repeat numbers of the respective marker in strain *L. *(*L.*) *major *MHOM/IL/1980/Friedlin.

### Data analysis

The software STRUCTURE Version 2 [16] was used to reveal potential population structure in the data set. The programme was run using an admixture model with a burn-in period of 30,000 iterations, followed by 1,000,000 Markov Chain Monte Carlo (MCMC) repeats. Based on allele frequencies, this approach identifies genetically distinct populations by estimating, for each individual studied, in this case each leishmanial strain, the fraction of the genotype belonging solely to it. Individuals can be assigned to multiple clusters with the membership coefficients of all those clusters summing up to one. The most fitting number of populations was determined by comparing log-likelihood values for K (number of populations) between 1 and 10. These were plotted and the value of K at the plateau (maximum) of the Gaussian graph plotted indicated the most likely population structure. In order to quantify the degree of variation of the likelihood for each K, ten runs were performed for each K. In addition, ΔK was calculated based on the rate of change in the log probability of data between successive K values [27]. Moreover, runs were conducted with strains assigned to 5 pre-defined populations (Central Asia, the Middle East, North Africa, East Africa and West Africa), according to the actual geographical origin of the CL isolates.

Different microsatellite genetic distance measurements based on the infinite allele model (IAM) and on the stepwise mutation model (SMM) were used to construct phylogenetic trees. The proportion of shared alleles (Dps) [28] calculates multilocus pairwise distance measurements as 1 – (the total number of shared alleles at all loci/n), where *n *is the number of loci compared. Chord distance [29], Nei's standard genetic distance [30] and delta mu squared (Dmu2) [31], were also calculated. The last is based on the average squared difference in allele size. Confidence intervals for all distance measurements were calculated by bootstrapping over loci, using the program MICROSAT [32]. Neighbor-joining (NJ) and Unweighted Pair Group Method with Arithmetic Mean (UPGMA) consensus trees of both distances were then constructed in PAUP, version 4.0b8 [33] and *PHYLIP *version 3.6 [34].

The degree of genetic difference among populations was estimated by Wright's F-statistics [35], calculated according to Weir and Cockerham [36]. FSTAT software version 2.9.3.2 [37] was used to calculate *Fis *and *Fst *values, numbers of alleles per locus and genetic flow or migration rate, as Nm = 1-*Fst*/4*Fst *[38]. Average sample size (n), mean number of alleles per locus (*A*), observed (*H*_*o*_) and expected (*H*_*e*_) heterozygosity under Hardy-Weinberg equilibrium were calculated using Gene Data Analysis (GDA), version 1.0 (d16c) [39]. The calculation was based on the permutation method [40], which is useful for multiallelic microsatellite loci. Pair-wise linkage disequilibrium (D) was calculated using GENEPOP and FSTAT. The former applies Fisher's exact test [41] to the allele combinations between all pairs of loci in the whole population and in each population, while the latter uses log-likelihood ratio G-statistics [36] with Bonferroni corrections for multiple comparisons.

To compare the allelic abundance in different geographical regions, the MLMT profiles of *L. *(*L.*) *major *were grouped according to their main geographical origin: Africa, Central Asia and Middle East. Profiles shared by more than one strain were excluded from this analysis. To compare the different groups, all of them were reduced to the size of the smallest group, which was Central Asia with 12 MLMT profiles. The groups of larger size were re-sampled and the number of alleles in each group was estimated, using 100 replicates of the re-sampled data [15].

## Authors' contributions

AA–J was involved in strain collection in ME, performed MLMT for the majority of the strains and analyzed the data. SD and MM designed the microsatellite markers and developed the MLMT protocol. TW supervised all population genetic aspects in this study. LFS collected and provided strains from ME. MVS, DAK and SAR provided the strains collected during different field trips in CA. KK and JS contributed new analytical tools. GS designed the research. The paper was written by GS, AA–J and LFS with the help of all co-authors. All authors have read and approved the final manuscript.

## Supplementary Material

Additional file 1 **Table S1:** Multilocus microsatellite profiles represented by the repeat numbers obtained for the markers, of the strains of L. (L.) major analysed in this study. Homozygote allele combinations are given as single numbers, two different numbers indicate heterozygote allele combinations and missing data are shown as --. Click here for file
