# Supplementary material for: Identification of geographically distributed sub-populations of Leishmania (Leishmania) major by microsatellite analysis
Source: BMC Evol Biol. 2008 Jun 24;8:183. doi: 10.1186/1471-2148-8-183 (PMC2447845; doi:10.1186/1471-2148-8-183)
Supplement: Additional file 1 — Table S1: Multilocus microsatellite profiles represented by the repeat numbers obtained for the markers, of the strains of L. (L.) major analysed in this study. Homozygote allele combinations are given as single numbers, two different numbers indicate heterozygote allele combinations and missing data are shown as --. [file 1471-2148-8-183-S1.pdf]

Table S1: Multilocus microsatellite profiles represented by the repeat numbers obtained for the markers, of the strains of *L.* (*L.*) *major* analysed in this study. Homozygote allele combinations are given as single numbers, two different numbers indicate heterozygote allele combinations and missing data are shown as --.

MLMT profile	Cluster	Repeat numbers for 10 microsatellite markers									
		4 GTG	27 GTG	36 GTG	39 GTG	45 GTG	1 GC	28 AT	71 AT	1 GACA	1 CA
Lmj 01	CA1	7	8	10	2	4	7	9	10	6	14
Lmj 02	CA1	7	8	--	2	4	7	9	10	6	14
Lmj 03	CA1	7	8	10	2	4	7	8/10	10	6	14
Lmj 04	CA1	7	8	10	2	4	7	9	10/11	6	14
Lmj 05	CA1	6	8	10	2	4	7	10	13	6	14
Lmj 06	CA1	7	8	10	2	4	7	10	13	6	14
Lmj 07	CA1	7	8	10	2	4	7	9	13/16	6	14
Lmj 08	CA1	7	8	10	2	4	7	9	13	6	14
Lmj 09	CA1	7	8	9	2	4	7	9	--	6	14
Lmj 11	CA2	7	8	9	2	4	7	9/10	13	6/7	14
Lmj 12	CA2	7	8	9	2	4	7	9	13/16	6	14
Lmj 13	CA2	7	8	9	2	4	7	--	16	6	14
Lmj 14	CA2	7	8	9	--	5	7	9	11	6	14
Lmj 15	CA2	7	8	9	2	4	7	9	13	6	14
Lmj 16	AF1	6	9	7	3	10	8	10	9	7	23
Lmj 17	AF1	6	9	7	3	10	7	13	13	7	17
Lmj 18	AF1	6	9	9	2	10	7	10	13	7	28
Lmj 19	AF1	6	9	9	2	10	7	10	13	8	28
Lmj 20	AF1	6	9	6	2	10	7	12	13	7	18
Lmj 21	AF1	6	9	7	3	10	7	13	7	7	17
Lmj 22	AF1	7	9	6	8	10	8	11	11	7	22
Lmj 23	AF1	7	9	6	8	10	8	10	13	7	22
Lmj 24	AF1	6	9	6	8	10	8	10	12	7	23
Lmj 25	AF2	6	10	--	--	18	--	8	9	6	--
Lmj 26	AF2	6	10	9	--	18	7	8	10	6	14
Lmj 27	AF2	6	10	9	--	18	7	5/8	9/12	6	14
Lmj 28	AF2	6	10	9	--	18	7	8	12	6	14
Lmj 29	AF2	6	10	7	10	18	8	10	11	7	17
Lmj 30	AF2	6	10	7	10	18	8	10	13	7	17
Lmj 31	AF2	6	10	7	10	18	7	5	14	8	8
Lmj 32	AF2	6	10	7	10	14	7	10	13	8	4
Lmj 33	AF2	6	10	8	10	18	7	9	15	8	8
Lmj 34	AF2	6	10	9	--	18	7	11	12	6	14
Lmj 35	AF2	6	10	7	10	19	7	11	15	8	8
Lmj 36	AF2	6	10	7	10	18	8	10	15	7	22
Lmj 37	AF1	6	9	6	12	9	7	16	13	6	24
Lmj 38	AF1	7	9	6	12	10	7	16	16	6	23
Lmj 39	ME1	7	9	9	9	12	7	9	13	7	14
Lmj 40	ME1	7	9	10	9	12	7	9	13	7	14
Lmj 41	ME1	7	7	9	9	12	7	9	13/16	7	14
Lmj 42	ME1	7	9	10	9	12	7	9	13	6	14
Lmj 43	ME1	7	9	9	12	12	7	9	13	7	14
Lmj 44	ME1	7	9	9	9	12	7	9	13/16	7	14
Lmj 45	ME1	7	9	9	9	12	7	10	16	7	14
Lmj 46	ME1	7	9	9	9	9	7	9	13	7	14
Lmj 47	ME1	7	9	10	9	9	7	9	11	7	14
Lmj 48	ME1	7	9	8	9	12	7	9	13	7	14
Lmj 49	ME1	7	9	8	9	12	7	8/9	13	7	14
Lmj 50	ME1	7/5	9	9	9	12	7	9	13	7	14
Lmj 51	ME1	7	9	9	9	12	6	9	13	7	14
Lmj 52	ME2	7	9	8	12	7/9	7	9	13	7	14

Lmj 53	ME2	7	9	9	12	9	8	9	13	7	19
Lmj 54	ME2	7	9	9	12	9	7	9	13	7	19
Lmj 55	ME2	7	9	9	12/14	12	8	9	13	7	19
Lmj 56	ME2	7	9	--	12	9	8	9	13	7	19
Lmj 57	ME2	7	9	9	12	10	8	9	13/16	7	19
Lmj 58	ME2	7	9	8	12	8	8	10	8	7	19
Lmj 59	ME2	7	8	9	12	12	8	9	16	7	19
Lmj 60	ME2	7	9	5	12	9	8	9	13	7	19
Lmj 61	ME2	7	9	5	12	9	8	9	16	7	19
Lmj 62	ME2	7	9	8	12	9	8	9	13	7	19
Lmj 63	ME2	7	9	8	12	9	8	9	14	6	19
Lmj 64	ME2	7	9	8	12	9	8	9	11	7	19
Lmj 65	ME1	7	6	5	12	9	7	9	13	6	14
Lmj 66	ME1	7	6	7	12	9	7	9	13	6	14
